# Generation of Long-Lived Bone Marrow Plasma Cells Secreting Antibodies Specific for the HIV-1 gp41 Membrane-Proximal External Region in the Absence of Polyreactivity

**DOI:** 10.1128/JVI.01089-16

**Published:** 2016-09-12

**Authors:** Luke R. Donius, Yuxing Cheng, Jaewon Choi, Zhen-Yu J. Sun, Melissa Hanson, Michael Zhang, Todd M. Gierahn, Susanna Marquez, Mohammed Uduman, Steven H. Kleinstein, Darrell Irvine, J. Christopher Love, Ellis L. Reinherz, Mikyung Kim

**Affiliations:** a Laboratory of Immunobiology and Department of Medical Oncology, Dana-Farber Cancer Institute, Boston, Massachusetts, USA; b Department of Biological Chemistry & Molecular Pharmacology, Harvard Medical School, Boston, Massachusetts, USA; c Departments of Materials Science and Engineering and Biological Engineering, Massachusetts Institute of Technology, Cambridge, Massachusetts, USA; d Department of Pathology, Yale School of Medicine, New Haven, Connecticut, USA; e Department of Immunology, Yale School of Medicine, and Interdepartmental Program in Computational Biology and Bioinformatics, Yale University, New Haven, Connecticut, USA; f Koch Institute for Integrative Cancer Research at MIT, Cambridge, Massachusetts, USA; g Howard Hughes Medical Institute, Chevy Chase, Maryland, USA; h Department of Dermatology, Harvard Medical School, Boston, Massachusetts, USA; Ulm University Medical Center

## Abstract

An effective preventive vaccine is highly sought after in order to stem the current HIV-1 pandemic. Both conservation of contiguous gp41 membrane-proximal external region (MPER) amino acid sequences across HIV-1 clades and the ability of anti-MPER broadly neutralizing antibodies (BNAbs) to block viral hemifusion/fusion establish the MPER as a prime vaccination target. In earlier studies, we described the development of an MPER vaccine formulation that takes advantage of liposomes to array the MPER on a lipid bilayer surface, paralleling its native configuration on the virus membrane while also incorporating molecular adjuvant and CD4 T cell epitope cargo. Here we demonstrate that several immunizations with MPER/liposomes induce high levels of bone marrow long-lived plasma cell (LLPC) antibody production. Single-cell immunoglobulin gene retrieval analysis shows that these plasma cells are derived from a germ line repertoire of B cells with a diverse representation of immunoglobulin genes, exhibiting antigen-driven positive selection. Characterization of LLPC recombinant monoclonal antibodies (rMAbs) indicates that antigen recognition is achieved through convergence on a common epitopic focus by utilizing various complementarity-determining region H3 (CDRH3) lengths. Importantly, the vast majority of rMAbs produced from these cells lack polyreactivity yet manifest antigen specificity in the context of lipids, shaping MPER-specific paratopes through selective pressure. Taken together, these findings demonstrate that the MPER is a vaccine target with minimal risk of generating off-target autoimmunity.

**IMPORTANCE** A useful vaccine must generate desired long-term, antigen-specific antibody responses devoid of polyreactivity or autoreactivity. The common polyreactive features of some HIV-1 BNAbs have raised concern about elicitation of anti-MPER antibodies. Utilizing single-LLPC repertoire analysis and biophysical characterization of anti-MPER rMAbs, we show that their fine specificities require a structural fitness of the antibody combining site involving heavy and light chain variable domains shaped by somatic hypermutation and affinity maturation of B cells in the germinal center. Perhaps more importantly, our results demonstrate that the majority of MPER-specific antibodies are not inherently polyspecific and/or autoreactive, suggesting that polyreactivity of MPER-specific antibodies is separable from their antigen specificity.

## INTRODUCTION

To date, no widely applicable cure for HIV-1 is known, and current preventive efforts have not proven completely effective. Successful vaccination would be a powerful means to fight the global HIV-1 pandemic. Unlike infectious diseases against which vaccines induce highly protective immunity (1), broad and potent neutralization of HIV-1 strains has not been elicited through vaccination with HIV-1 protein envelope (Env) subunits or inactivated virus. However, the discovery of numerous broadly neutralizing antibodies (BNAbs) capable of blocking viral binding to or entry into host cells suggested that vaccination is a promising strategy (2–4).

The HIV-1 envelope spike protein, comprised of trimeric gp41 and gp120 subunits, is the only viral target exposed on the virion membrane surface and therefore is the singular focus for an antibody-based vaccine. The first HIV-1 BNAb discovered, 2F5, is specific for the membrane-proximal external region (MPER), and more recently, the MPER-specific neutralizing antibody list has grown to include 4E10, Z13e1, m66, m66.6, 10E8, and CAP206-CH12 (5–12). The BNAb list has also widened over time with the identification of a variety of other targets, including the CD4-binding site, the V1/V2-glycan-containing epitope, the V3-glycan-containing epitope, and gp120/gp41-bridging epitopes. These BNAbs were discovered through the recovery of single memory B cells from infected individuals and by recombinant monoclonal antibody (rMAb) production (reviewed in references 2, 13, and 14). Nevertheless, as one of the most highly conserved regions on the envelope spike, the MPER remains an exemplary vaccine target (9, 15, 16).

The MPER is a hydrophobic and tryptophan-rich segment of 22 amino acids located immediately external to the transmembrane (TM) domain of gp41 (15, 17). Structurally, the MPER consists of two alpha-helices connected by a linker in a helix-hinge-helix motif in a lipid environment (16, 18). We previously showed that the BNAbs 2F5 and 4E10 mediate extraction of their epitopic residues on the MPER helices from the lipid membrane (18–20). Very recently, the first micelle-embedded trimer spike structure that includes the MPER and TM regions was elegantly solved using cryo-electron microscopy (cryo-EM), and this structure suggests that in a 10E8-bound conformation, the MPER is lifted up off the membrane (21). A recent crystallographic analysis identified a lipid as an integral component of the 4E10 BNAb and implied a similar MPER segment extraction geometry out of the membrane (22). Functionally, the MPER has been shown to be required for both hemifusion and fusion processes preceding viral entry (15–17, 23–25), presumably through its strong interaction with the membrane. Therefore, antibodies elicited by vaccination that bind with high affinity to the MPER on the trimer would impede or block MPER function and manifest neutralizing activity.

Extensive biochemical and structural analyses of MPER-specific BNAbs have suggested the obligate role of the membrane environment in MPER immunogen design, both to configure native MPER structure and to induce potent BNAbs (18, 19, 22, 26–35). Such requirements are likely explanations for the lack of anti-MPER neutralizing antibodies elicited through vaccination with free MPER peptides, MPER epitope mimetics, or MPER epitopes grafted onto protein scaffolds (28, 36–38; reviewed in reference 39). Nonetheless, while liposome-based MPER vaccines induce strong MPER antibody responses (40–45), the chemical modifications used for membrane anchoring and prevention of proteolysis, such as palmitic acid adducts and amide capping, respectively, have an impact on antigenic determinants even without inducing alterations in the MPER segment structure (42). These challenges for membrane-arrayed MPER segment immunogen design still await innovative solutions. Moreover, it has been proposed that sequence similarity of endogenous mammalian proteins to the MPER limits expansion of B cells expressing germ line-encoded MPER-reactive B cell receptors (BCR) because of negative selection during development (46, 47). It has further been suggested that HIV-1 Env gp41 antibodies might arise from polyreactive B cells, possibly from the pool of gut microbe-regulating B cells (48, 49). This notion might explain why acutely HIV-1-infected subjects have antibodies whose unmutated ancestors react with bacterial or host antigens but not with the HIV-1 envelope. The highly polyreactive nature of the MPER BNAbs 2F5 and 4E10 has been construed as evidence for the existence of such a pathway (47, 50, 51). These are sound proposals; however, given the ability of certain BNAbs, notably 10E8, to arise independently of polyreactivity (6), it is not a *sine qua non* that a successful MPER immunogen must elicit antibodies with this characteristic in order to be broadly neutralizing.

In this study, we experimentally assessed the extent to which durable, affinity-matured anti-MPER antibody responses induced by MPER/liposome vaccination exhibit polyreactivity. We demonstrated that MPER/liposome immunizations generate MPER-specific bone marrow (BM) long-lived plasma cell (LLPC)-derived class-switched Abs. Single-plasma-cell analysis followed by immunoglobulin gene rescue and sequencing and analysis of expressed rMAbs revealed that BM LLPC use a diverse collection of immunoglobulin genes with the same epitope specificity, but with functionally distinct characteristics. Affinity maturation of multiple distinct B cells postimmunization and -boosting yielded MPER epitope specificity through structural fitness and convergence on a common antigenic determinant, accompanied by little, if any, polyreactivity for 39 of 44 rMAbs analyzed. These characteristics demonstrate that MPER specificity is not defined by polyreactivity for the vast majority of rMAbs elicited by vaccination that recognize membrane-embedded MPER.

## MATERIALS AND METHODS

### Mice and MPER/liposome immunizations.

BALB/c mice were obtained from Taconic Biosciences (Hudson, NY). All mice used were 8 to 10 weeks of age at the time of initial immunization. Mice were housed in a specific-pathogen-free facility and maintained in accordance with procedures and protocols approved by the Dana-Farber Cancer Institute and Harvard Medical School Animal Care and Use Committee Institutional Review Board. Immunization liposomes were made as described before (42) by drying the following components under a nitrogen stream and placing them under vacuum overnight: N- or C-terminally palmitoylated MPER peptides (Npalm-MPER or Cpalm-MPER), monophosphoryl lipid A (MPLA) from Salmonella enterica serotype Minnesota (Sigma-Aldrich, St. Louis, MO), and the lipids 1,2-dioleoyl-*sn*-glycero-3-phosphocholine (DOPC), 1,2-dimyristoyl-*sn*-glycero-3-phosphocholine (DMPC), and 1,2-dioleoyl-*sn*-glycero-3-phospho-(1′-*rac*-glycerol) (DOPG) (Avanti Polar Lipids Inc., Alabaster, AL) at a 2:2:1 ratio. Liposomes were formed with encapsulated LACK1 peptide by rehydration with phosphate-buffered saline (PBS) containing 1,000 µg/ml LACK1 to a final total liposome component of 25.2 mg/ml. Final liposomes incorporated MPER at a 1:200 molar ratio (MPER:lipid) and contained 175 μg/ml MPLA, with a total lipid concentration of 25 mg/ml. MPER/liposomes were sized by vortexing 6 times for 30 s each at 5-min intervals, 6 rounds of flash freezing in liquid nitrogen and thawing at 37°C, and extrusion by passage through a 100-nm-pore-size polycarbonate membrane 21 times. Mice were immunized intradermally with 50 μl per hind flank. Unless noted otherwise, immunizations were administered on three occasions at 21-day intervals.

### Microengraving and single-B-cell isolation.

Npalm-MPER/liposomes, consisting of MPER at a 1:50 molar ratio with DOPC and DOPG lipids (4:1) at a final total liposome component of 2 mg/ml, were rehydrated by vortexing, freeze-thawing, and extrusion as described above. Polylysine-coated glass slides (25 × 75 × 1 mm) were incubated overnight with rocking at room temperature, submerged in 100 μg/ml liposomes in PBS. The liposome solution was poured off, and slides were blocked for 1 h in a 3% milk-0.05% Tween 20-PBS solution with rocking at room temperature. Polymethylsiloxane microwell molds were prepared as described in detail by Ogunniyi et al. (52). Molds were subjected to plasma cleaning under vacuum to charge and sterilize the surface and then were blocked with 1% bovine serum albumin (BSA) in PBS for 20 min. The blocking solution was rinsed from slides with purified water. CD138^+^ plasma cells were sorted from the BM of femurs and tibias from one mouse by use of a Miltenyi Biotec (Bergisch Gladbach, Germany) magnetic bead labeling and enrichment kit. The mold was removed from blocking buffer and the liquid aspirated, followed by three washes with 10% fetal bovine serum containing 1% penicillin-streptomycin in RPMI 1640 (growth medium). The growth medium was aspirated, and plasma cells (∼100,000 in 300 μl growth medium) were distributed dropwise evenly across the mold. The growth medium in the mold was aspirated, and growth medium was distributed onto the mold surface. The medium was then aspirated, and growth medium plus 100 ng/ml interleukin-6 (IL-6) was distributed on the mold before the MPER/liposome-labeled slide was placed on the mold and clamped in place in a hybridization chamber for 1 h at 37°C. Following incubation, the slide was washed and blocked, and secondary antibody (goat anti-mouse IgG–AF647 and anti-IL-6–AF488) hybridization was carried out in a Tecan HS 400 Pro slide washer (Tecan, Mannedorf, Switzerland). Microarray analysis of slides was performed using a GenePix 4400 instrument (Molecular Devices LLC, Sunnyvale, CA) at 488 nm and 635 nm, and identification of wells containing MPER-specific cells was performed using GenePix Pro 6 analysis software. The mold with plasma cells was stored submerged in growth medium at 4°C overnight, and single plasma cells were isolated by use of a micromanipulator and placed into 96-well plates containing a solution of 1 μl RNasin Plus RNase inhibitor (Promega, Madison, WI) and 9 μl UltraPure DNase-/RNase-free H_2_O (Life Technologies, Grand Island, NY). Plates were sealed and stored at −80°C for later RNA isolation/cDNA synthesis.

### MPER-specific single-B-cell cDNA generation, PCR, and cloning.

Our method for cloning and expression of mouse immunoglobulin genes from single B cells was adopted from the method developed by Tiller et al. (53) and utilized the primers and vectors they described. In brief, 96-well plates with single B cells were thawed on ice, and 5 μl of reverse transcription primer mix (1 μl random primers, 1 μl deoxynucleoside triphosphates [dNTPs] [10 mM concentration of each], 1 pmol [each] 1st-round PCR reverse heavy and kappa light chain [IgH and IgLk] primers, and DNase/RNase-free water) was added to each well. The plate was heated to 65°C for 5 min in a thermocycler and then cooled on ice for 1 min. SuperScript3 reverse transcription mix containing 5× reverse transcription buffer, dithiothreitol (DTT), RNase Out, SuperScript3, and DNase/RNase-free water was added to a final volume of 30 μl, and cDNA synthesis was performed by running the following ramped thermocycler program: 25°C for 5 min, 50°C for 60 min, 55°C for 60 min, and 70°C for 15 min. A nested PCR strategy was used to amplify the IgH or IgLk immunoglobulin gene from cDNA. First- and second-round nested PCRs were performed using high-fidelity Q5 polymerase (New England BioLabs, Ipswich, MA). For IgH amplification, 3 μl of cDNA reaction mix was added to a total of 50 μl of PCR mix and amplified using the following program: 98°C for 30 s, 50 cycles of 98°C for 30 s, 50°C for 30 s, and 72°C for 30 s, and a final extension at 72°C for 10 min. The same strategy was implemented for IgLk amplification, but with a 47°C annealing temperature instead of 50°C. The second-round PCR was performed using 1 μl of the first-round template for nested amplification with the same thermocycler programs as those described above. DNA sequences were obtained using the second-round PCR 5′ primer mix. IgH and IgLk pairs of interest were cloned by determining the closest-matching V-gene/J-gene cloning primer sets described by Tiller et al. and reamplifying the sequence from the first-round PCR product. The resulting fragment was gel purified, inserted into the pCR-BLUNT II TOPO vector by use of topoisomerase I (Zero Blunt TOPO cloning kit; Thermo Fisher, Waltham, MA), and transfected into Escherichia coli for subcloning (One-Shot TOP10 chemically competent cells; Thermo Fisher, Waltham, MA). Plasmids were isolated, sequenced to confirm fidelity, and restriction digested for insertion into the IgH and IgLk expression vectors for human IgG1 and kappa chain, respectively. Expression of rMAbs was performed by transfecting 30-ml cultures of exponential-growth-phase 293F cells with 20 μg each of IgH and IgLk in their respective vectors by use of 293Fectin (Thermo Fisher, Waltham, MA) with shaking in a cell culture incubator at 37°C and 5% CO_2_ for 7 days. Expressed rMAbs were isolated by pelleting cells, filtering the supernatants to 0.22 μm, purifying immunoglobulin by use of Gamma-bind Plus Sepharose beads (GE Healthcare, Little Chalfont, United Kingdom), and concentrating the samples with 30K centrifugal filters.

### MPER/liposome and cardiolipin/dsDNA ELISAs.

Enzyme-linked immunosorbent assays (ELISAs) for measuring MPER-specific antibodies from sera were done by labeling Immulon 1B plates overnight with 2 μg/ml streptavidin in PBS (50 μl/well) at 4°C. The following day, plates were washed three times with 0.1% BSA-PBS and blocked with 100 μl per well 1% BSA-PBS for 3 h. Incubation with 0.2% biotinylated polyethylene glycol (PEG) 2000-containing Npalm-MPER-NH_2_/liposomes or Npalm-MPER-COOH/liposomes (1:50 peptide:lipid ratio) at 32 μg/ml in 1% BSA-PBS was then carried out for 2 h with shaking at room temperature and then for another 2 h at 4°C. Plates were then washed again, and serially diluted sera in 1% BSA-PBS were aliquoted and incubated overnight with gentle rocking at 4°C. The following day, serum samples were removed, the plate was washed, and goat anti-mouse–horseradish peroxidase (HRP) secondary antibody (Bio-Rad, Hercules, CA) at a 1:2,000 dilution was applied for 1 h at 4°C. Plates were washed two times with 0.1% BSA-PBS and two times with PBS. Bound antibody was detected by incubation with *o*-phenylenediamine (OPD) solution in citrate buffer, pH 4.5, for 10 min. The OPD reaction was stopped with 2.25 M H_2_SO_4_, and the absorbance was read at 490 nm on a Victor X4 plate reader (Perkin-Elmer, Waltham, MA). Autoreactivity/polyreactivity of MPER-specific rMAbs was tested by ELISAs with cardiolipin and double-stranded DNA (dsDNA) by coating Immulon 2HB plates (Thermo Scientific, Waltham, MA) with 75 μg/ml cardiolipin (Sigma-Aldrich, St. Louis, MO) in ethanol and allowing them to air dry overnight or coating them with 100 μg/ml of sheared 0.45-μm-filtered salmon sperm DNA (Life Technologies, Grand Island, NY) in PBS overnight at 4°C. Plates were washed three times with 0.05% Tween 20-PBS, and all wells were blocked with 1% BSA-PBS for 1 h at room temperature with shaking. Plates were washed with 0.05% Tween 20-PBS, and serially diluted rMAbs were incubated for 1 h at room temperature. Washing was repeated, and the wells were incubated with 1:3,000 goat anti-human IgG–HRP (Bio-Rad, Hercules, CA) antibody for 1 h at room temperature. Plates were washed six times with 0.05% Tween 20-PBS, and bound antibody was detected using OPD solution as described above. Non-antigen-coated plates were set up in parallel to measure nonspecific binding and to subtract the background signal from the total.

### ELISPOT quantification of MPER-specific ASCs.

Enzyme-linked immunosorbent spot (ELISPOT) analysis for quantifying the numbers of antigen-specific antibody-secreting cells (ASCs) from BM, spleens, and lymph nodes was performed using 96-well 0.45-μm, hydrophobic, high-protein-binding Immobilon-P polyvinylidene difluoride (PVDF) membrane plates (EMD Millipore, Billerica, MA). Plates were activated with 35% ethanol and then washed eight times with water, followed by incubation with 100 μl per well of various 100-μg/ml Npalm-MPER/liposome formulations in PBS (1:50 or 1:1,000 MPER:lipid ratio; 4:1 DOPC:DOPG ratio) overnight at 4°C. Plates were washed six times with 0.1% BSA-PBS, blocked with 200 μl/well 1% BSA-PBS for at least 4 h, washed once with RPMI 1640 medium supplemented with 10% FBS, glutamine, 2-mercaptoethanol, and penicillin-streptomycin, and then blocked for 1 h at 37°C with the same medium. Meanwhile, mouse spleens, inguinal lymph nodes (iLN), and BM were isolated, and single-cell suspensions were prepared in the aforementioned growth medium. Cells were strained to 70 μm, quantified using a hemacytometer, and resuspended to 1 × 10^7^ cells/ml. The growth medium block was removed from plates and replaced with 50 μl/well fresh growth medium. Cell suspensions were restrained to 70 μm and added to wells in 50-μl volumes (500,000 cells), in triplicate or quadruplicate. Hybridoma cells (M1) which secrete a C-terminal MPER-specific antibody and BNAb 2F5-expressing cells were plated as controls. Cells were incubated overnight at 37°C and 5% CO_2_ in a humidified chamber. The following day, wells were washed six times with 0.1% BSA-PBS and blocked for 1.5 h with 1% BSA-PBS. The blocking buffer was discarded, and bound antibody was detected using 0.6 μg/ml of alkaline phosphatase (AP)-conjugated goat anti-mouse secondary antibodies (anti-IgM, anti-IgG1, anti-IgG2a, anti-IgG2b, anti-IgG3, and anti-IgG; Jackson ImmunoResearch, West Grove, PA) in 1% BSA-PBS. BNAb 2F5-secreting control cells were visualized using goat anti-human IgG–AP. To visualize MPER-specific bound antibody, wells were washed eight times, and 100 μl/well 5-bromo-4-chloro-3-indolyl phosphate/nitro blue tetrazolium (BCIP/NBT) solution was added for 5 min. Plates were washed thoroughly with distilled water and dried overnight. Spots were quantified using a CTL ImmunoSpot ELISPOT plate reader and ImmunoSpot 3 software (CTL, Shaker Heights, OH).

### SPR analysis.

Surface plasmon resonance (SPR) analysis was done as described previously (42). Briefly, for alanine scanning analysis, 30 μl of 150 μM-250 μM DOPC-DOPG liposomes in running buffer (20 mM HEPES, 0.15 mM NaCl, pH 7.4) was applied to a Pioneer L1 sensor chip in a BIAcore 3000 instrument at a flow rate of 3 μl/min at 25°C. Multilamellar structures were removed by injection of 20 μl of 25 mM sodium hydroxide at a flow rate of 100 μl/min. MPER peptides (0.5 μM) were dissolved in running buffer right before injection and complexed with the liposomes by injection of 60 μl at a flow rate of 10 μl/min. Binding of MAb was then tested by passage of the MAb over the peptide-liposome complex at 10 μl/min. Peptide-liposome complexes were removed by sequential passages of 40 mM 3-[(3-cholamidopropyl)-dimethylammonio]-1-propanesulfonate (CHAPS) and 3:2 sodium hydroxide (50 mM)-isopropyl alcohol over the sensor chip. For liposome-only or MPER/liposome rMAb binding analysis, 30 μl of each purified rMAb at 30 μg/ml was applied to the chip at a flow rate of 10 μl/min for 3 min, using a protocol identical to that for alanine scanning analysis (42).

### Immunoglobulin gene sequence analysis.

Immunoglobulin genes from two mice (*n* = 66 total sequences generated) were amplified from single MPER-specific IgG-secreting plasma cells. The sequences were aligned by use of IMGT/HighV-QUEST (version 1.5.1) to identify their V(D)J germ line segments. Sequences for which a junction could not be identified (*n* = 18) were excluded from further analyses, leaving 15 sequences for mouse 1 and 33 for mouse 2 for detailed investigation. Downstream analyses were performed using tools from the Change-O suite (version 0.3.3-2016.04.22) and the associated R packages SHazaM (shazam_0.1.2.999) and alakazam (alakazam_0.2.3.999) (54). Detailed information is available at http://immcantation.readthedocs.io. Sequences were assigned to the same clone when they shared the same IGHV gene, IGHJ gene, IGLkV gene, IGLkJ gene, and junction length, with a Hamming distance threshold of 0.05, using the DefineClones command line tool. For each clone, the germ line sequences were identified for the heavy and light chains by use of the CreateGermlines tool, and the germ line definitions were retrieved from IMGT (as of 2 May 2016). SHazaM was used to quantify the mutation frequencies for the V segment up to the start of CDR3 by comparison to the IMGT germ line gene segment, and also broken down by region (complementarity-determining region [CDR], framework region [FWR], or entire sequence). BASELINe (version 1.3 [30 January 2014]) (55) was used to quantify selection strength in the CDR and FWR by using the Focused test statistic and the mouse trinucleotide SHM targeting model and selecting the clonal sequence option.

### MPER constructs.

MPER peptides were generated on an ABI 431 peptide synthesizer by using Fmoc chemistry, high-pressure liquid chromatography (HPLC) purification, and postpurification conjugation of N-terminal palmitic acid at the Massachusetts Institute of Technology, as described previously (42). Peptide amino acid sequences were as follows: MPER-NH_2_, palm-ELDKWASLWNWFNITNWLWYIK-NH_2_; MPER-COOH, palm-ELDKWASLWNWFNITNWLWYIK-COOH; W680A-COOH, palm-ELDKWASLWNWFNITNWLAYIK-COOH; MPER-N, palm-GSGSDLLELDKWASLWNWFNIT-NH_2_; and MPER-C, palm-GGGSSASLWNWFNITNWLWYIK-NH_2_.

## RESULTS

### MPER/liposome immunizations generate antigen-specific LLPC.

**FIG 1:**
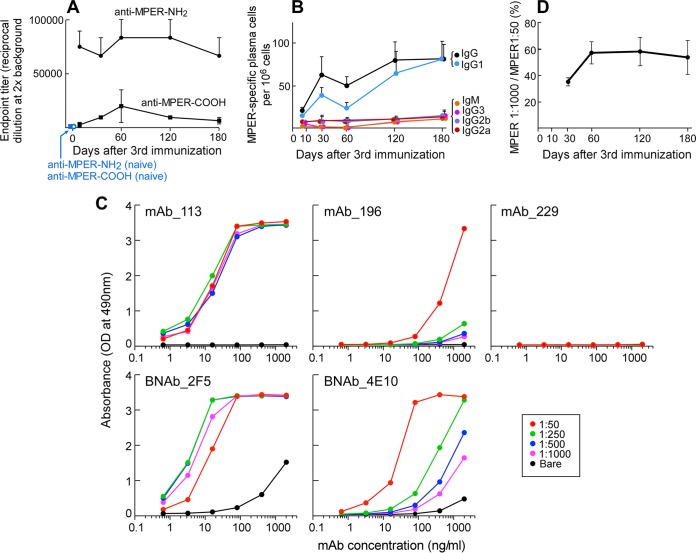
MPER/liposome immunization results in long-term, MPER-specific, isotype-switched antibody responses. (A) Endpoint titers of MPER-NH_2_/liposome (circles)- and MPER-COOH/liposome (squares)-specific IgGs were measured by ELISA at the indicated time points after the third immunization. Control data from naive mice are shown with blue circles (anti-MPER-NH_2_/liposomes) and squares (anti-MPER-COOH/liposomes). (B) Kinetics of MPER-binding and isotype-specific ASCs elicited after the third immunization. Numbers of MPER-binding ASCs from BM were determined by ELISPOT assay, using MPER-NH_2_/liposomes as the capture antigen. Quantification of numbers of anti-MPER-specific ASCs per 10^6^ bone marrow cells was performed by ELISPOT assay at the indicated time points. Secretion of immunoglobulin was assessed by specific isotype. Symbol colors: black, IgG; blue, IgG1; orange, IgM; pink, IgG3; purple, IG2b; red, IgG2a. (C) Low-affinity (196) but not high-affinity (113) rMAbs are sensitive to the copy number of MPER segments arrayed on the surface of a liposome. ELISA plates coated with streptavidin were incubated with liposomes containing biotin-PEG 2000 as well as MPER at peptide:lipid ratios of 1:50 (red), 1:250 (green), 1:500 (blue), and 1:1,000 (purple) or with bare liposomes (black). All serially diluted Abs (from 2 μg/ml to 64 pg/ml) were incubated on liposomes overnight and developed with anti-human secondary antibody. Points shown represent means for duplicate analyses. High-affinity BNAbs 2F5 and 4E10 were compared as positive controls, and irrelevant rMAb 229 was used as a negative control. (D) Relative affinities of MPER-binding IgG BM ASCs at different time points as assessed by ELISPOT assay. The frequencies of low-density MPER/liposome (1:1,000 [mol:mol])- and high-density MPER/liposome (1:50)-binding IgG ASCs were determined, and the ratios of low-density MPER (1:1,000) to high-density MPER (1:50) binding were plotted. All points in panels A, B, and D are means ± standard errors of the means (SEM) for quantification of data from 3 independent mice at each time point for ELISA and ELISPOT assays. Representative data are shown. All graphs represent BALB/c mice immunized with Npalm-MPER/liposomes loaded with a LACK1 T cell helper peptide and MPLA as an adjuvant, administered by intradermal injections on three occasions at 3-week intervals.

Circulating high-affinity antibodies are maintained for decades by LLPC without reexposure to antigen, providing protection against previously encountered pathogens (56, 57). Therefore, an effective antibody-based vaccine must stimulate production of antigen-specific LLPC. We tested the ability of the MPER/liposome formulation to generate LLPC and characterized this population at the single-cell level. To this end, BALB/c mice were immunized three times at 21-day intervals with the MPER anchored to the liposome membrane via a covalently attached N-terminal palmitoyl adduct. The liposomes were previously optimized (41, 42) to trigger humoral responses by including the Toll-like receptor 4 (TLR4) agonist MPLA as an adjuvant and the I-A^d^-presented T cell epitope LACK1, the immunodominant peptide of the Leishmania major LACK protein, to stimulate CD4 T cell help (58). Following immunization, sera were drawn from mice and endpoint titers for IgG isotype antibody binding to MPER/liposomes determined by direct ELISA. ELISA revealed that MPER-specific antibody endpoint titers of approximately 80,000 were maintained for up to 180 days following the third immunization (Fig. 1A). Since we previously showed that MPER/liposome immunizations result in dominant antibody responses directed to the C-terminal helix of the MPER, including the free amide exposed on the C-terminal end of the MPER sequence following synthesis (K683) (42), we also determined the magnitude of the serum antibody responses independent of the free amide in binding specificity by using MPER-COOH peptides. The analysis revealed that non-amide-reactive MPER-specific serum antibody responses were durably maintained by LLPC as well, albeit at 8 times lower titers.

Next, we determined the frequency of ASCs in BM over time following the third immunization. By coating 96-well PVDF membrane plates with MPER/liposomes and then incubating BM cells on the membranes overnight, the ASCs were visualized and quantified by ELISPOT assay, analogously to the procedures employed for soluble protein antigens. Consistent with the serum endpoint titer results, the majority of MPER-specific ASCs (50/10^6^ cells to 100/10^6^ cells) were of the switched IgG isotype, particularly IgG1; however, ∼10 MPER-specific ASCs/10^6^ cells of each of the IgM, IgG2a, IgG2b, and IgG3 isotypes were also maintained for up to 180 days (Fig. 1B). The same trend was also observed with MPER-specific BM plasma cells after the second immunization (data not shown). To assess the relative affinities of antibodies produced by ASCs in BM for the MPER following the third immunization, we developed a simple assay based on ligand display. We reasoned that a low-affinity antibody would not bind to MPER/liposomes in which the MPER was presented at a low density (1:1,000 peptide:lipid ratio) versus the higher density (1:50 ratio) used for total ASC quantification. The use of lower-density MPER in the liposome array (1:1,000 ratio) to exclusively detect high-affinity antibodies was validated by control experiments using representative high- and low-affinity rMAbs (113 and 196, respectively) elicited by MPER/liposome immunization and by comparison with the high-affinity 2F5 and 4E10 BNAbs (Fig. 1C). In addition, as shown subsequently, the relative binding affinities of rMAbs 113 and 196 were 3,400 response units (RU) and 380 RU, respectively, for MPER/liposomes at 30 μg/ml as measured by surface plasmon resonance (SPR) (see Fig. 4A). Parallel analysis at various MPER:lipid density ratios (1:50 to 1:1,000) by ELISA revealed that 2F5, 4E10, and the high-affinity clone 113 bound well. In contrast, the low-affinity clone 196 antibody bound to MPER/liposomes at the 1:50 ratio with a high signal but had little reactivity to those at ratios at or below 1:250 (Fig. 1C). Quantification of the numbers of cells binding the MPER/liposomes at 1:1,000 (low ratio) versus the high ratio (1:50) by ELISPOT analysis revealed that the percentage of cells binding both MPER/liposomes at 1:1,000 and MPER/liposomes at 1:50 reached and was maintained at nearly 60% by 60 days after the third immunization (Fig. 1D). These analyses indicate that the MPER-specific IgG-producing LLPC in BM are associated with the persistent MPER-specific antibody responses in serum. Moreover, not all MPER-specific LLPC in BM produce high-affinity Abs (e.g., rMAb clone 196 has a low affinity) (Fig. 1C).

### Isolation of single MPER-specific LLPC by microengraving.

**FIG 2:**
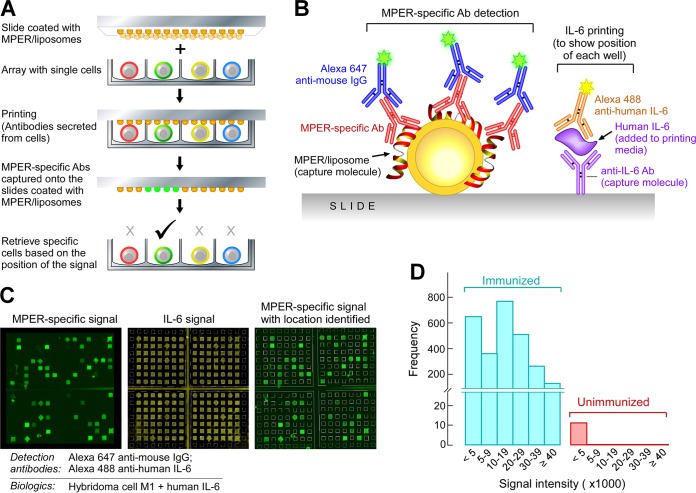
Identification and isolation of single MPER-specific BM plasma cells by use of microengraving technology. (A) Diagram of the microengraving method. (B) Interrogation of slides after printing. MPER-specific Abs captured on MPER/liposome-coated slides were detected with Alexa 647-conjugated anti-mouse IgG. Human IL-6 was detected with Alexa 488-conjugated anti-human IL-6 to produce signals in every well for grid alignment and localization of MPER-specific signals. Human IL-6 was added to the printing medium. (C) Establishment of the microengraving system by using MPER-specific M1 hybridoma cells. The MPER-specific signals (green squares) were overlaid on the IL-6 signals (yellow squares) for localization of MPER-specific cells and retrieval for analysis. The MPER-specific M1 hybridoma cell line was used as an example. (D) Detection of MPER-specific plasma cells from bone marrow. Plasma cells were isolated from BM by depleting B220^+^ cells and CD49b^+^ cells and then enriching for CD138^+^ cells. A total of 2,674 MPER-specific signals were detected from 70,000 BM plasma cells isolated from one mouse immunized with Npalm-MPER/liposomes, whereas only 11 signals, all with signal intensities of <5,000, were detected from an unimmunized mouse.

We next sought to characterize the variable (V), diversity (D), and joining (J) gene segments of immunoglobulins elicited by MPER/liposomes by using DNA sequencing at the single-cell level. This approach allowed us to determine the diversity of B cells recruited as components of the anti-MPER response by the extent to which these cells underwent somatic hypermutation (SHM) and then through recombinant protein expression to define the molecular characteristics, including CDR3 lengths, important for MPER binding. To identify and isolate MPER-specific LLPC, we took advantage of the versatile microengraving technology, which uses a dense array of microwells (0.1 to 1 nl each) containing individual cells to print a corresponding array of antibodies secreted by each cell (59, 60). Microengraving is performed by distributing cells on an injection mold containing grids of microwells and exposing the wells to an antigen-coated slide. In detail, plasma cells were purified from mouse BM cells 10 days after the third immunization with various MPER/liposome formulations, resuspended in medium containing human IL-6 to mark the grid, and distributed onto an array of microwells in a poly(dimethylsiloxane)-injected mold to allow the analysis of ∼100,000 total cells per array (Fig. 2A). A glass slide coated with MPER/liposomes (1:50 MPER:lipid ratio) and anti-human IL-6 was placed over the wells. The slide-mold arrangement was clamped into position and incubated for 1 h to allow secreted antibodies to bind to MPER/liposomes, while the IL-6 was bound by the coprinted anti-IL-6 capture antibody (Fig. 2B). M1 hybridoma cells producing MPER-specific antibody were tested as a positive control to demonstrate the lucidity of the method for localizing wells with MPER-specific cells (Fig. 2C, left panel). After incubation, the slide was removed, and Alexa 488-labeled anti-IL-6 was used to visualize the grid of the mold (Fig. 2C, middle panel, yellow wells). The MPER-specific antibody was detected with Alexa 647-labeled anti-mouse IgG, and this signal (shown in green) was overlaid on the IL-6 signal so that wells containing MPER-specific cells could be located (Fig. 2C, right panel). The cells were binned by their supernatants' MPER antibody detection signal intensities (Fig. 2D) and were retrieved by use of a micromanipulator for reverse transcription-PCR (RT-PCR).

The frequencies of MPER-specific plasma cells in BM 10 days after the third immunization with Npalm-MPER/liposomes ranged from ∼1.5 to 3% of total plasma cells by microengraving; the results of one representative experiment are shown in Fig. 2D. Only 10 to 20 cells from an immunologically naive mouse were scored positive, but with signal intensities of <5,000 (Fig. 2D).

### Genetic characterization of MPER-specific Ig repertoires.

**FIG 3:**
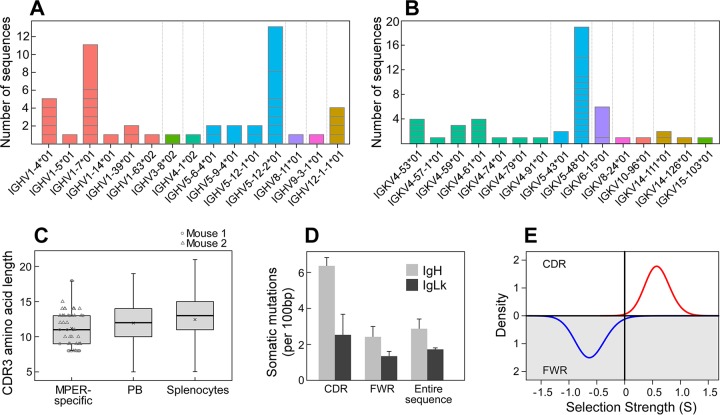
Immunoglobulin gene usage within the MPER-specific bone marrow plasma cell population is diverse. Immunoglobulin genes from two mice (*n* = 66 total sequences generated; 48 sequences were analyzed) were amplified from single MPER-specific IgG-secreting plasma cells. The usage frequencies of immunoglobulin V_H_ genes (IGHV) (A) and V_L_ genes (IGKV) (B) are diverse. V gene families were defined using IMGT and color coded by gene family. The number of sequences representing each IGHV and IGKV family is shown, with horizontal gray lines separating groups of clonally related sequences within each V gene cohort. (C) Box plot of CDRH3 length distributions. CDRH3 regions were identified by IMGT, and amino acid lengths were calculated. Each point represents a sequence from mouse 1 (circles) or mouse 2 (triangles). For comparison, mouse CDRH3 lengths for IgG sequences from splenocytes or plasmablasts (PB) are shown, using previously published data (76). Note that a single outlier point for mouse splenocytes is not shown. The mean value for each group is marked with a cross. (D) The number of somatic mutations per 100 germ line-defined base pairs was calculated for the CDRs, the FWRs, and the entire sequence of the IgH (gray columns) and IgLk (black columns) V genes. (E) BASELINe analysis of selection pressure on IgH V gene sequences. Significant positive selection was calculated for nonsynonymous CDR mutations (Σ = 0.59; *P* = 0.005; red line), along with significant negative selection against nonsynonymous mutations in the FWR region (Σ = −0.62; *P* = 0.015; blue line). The data represent analyses of sequences from two independent B cell/immunoglobulin gene isolations by microengraving.

BNAbs to HIV have been typified by long CDRH3 sequences and high mutation frequencies (6, 61), with the antibody responses to some epitopes dominated by particular VH genes, such as the well-defined prevalence of VH1-2 among the CD4-binding site agonist antibodies (62–65). To investigate the genetic composition of the MPER-specific Ig repertoires, RT-PCR was utilized to amplify variable regions of naturally paired IgH and IgLk antibodies by using a strategy previously described by Tiller et al. (53). Immunoglobulin genes from two mice immunized with Npalm-MPER/liposomes were amplified from single MPER-specific IgG-secreting plasma cells. The sequences (*n* = 66 total sequences generated) were aligned by use of IMGT/HighV-QUEST (version 1.5.1) to identify their V(D)J germ line segments. Sequences for which a junction could not be identified (*n* = 18) were excluded from further analyses, leaving 15 sequences for mouse 1 and 33 for mouse 2. The MPER-specific immunoglobulin repertoires were derived from a diverse collection of gene families for both the V_H_ (IgH V gene) (Fig. 3A) and V_L_ (IgLk V gene) regions (Fig. 3B), some of which were clonally expanded. As shown in Fig. 3C, the sequences also used a diverse set of CDRH3 lengths, which fell within the range of expected lengths for mice. Virtually all of the sequences (94%) showed somatic hypermutation, with averages of 2.9 and 1.7 mutations/100 bases for V_H_ and V_L_, respectively, and with a higher frequency of mutations in the CDRs (Fig. 3D). To determine whether these mutation patterns were driven by affinity maturation, we quantified selection strength (Σ) by using the BASELINe method (55). Positive values for Σ reflect a higher incidence of nonsynonymous mutations than expected and are associated with positive selective pressure, while negative values for Σ reflect a higher incidence of synonymous mutations than expected and are associated with negative selection pressure. BASELINe analysis revealed significant evidence for positive selection in the CDR, as expected for antigen-driven affinity maturation (Σ = 0.59; *P* = 0.005). In addition, there was significant negative selection in the framework regions (FWR), as expected to preserve the overall antibody structure (Σ = −0.62; *P* = 0.015) (Fig. 3E). Thus, the MPER-specific Ig repertoires reflect a diverse, affinity-matured cell population.

### Residue-specific binding analysis of rMAbs reveals common epitope recognition with diverse functional characteristics.

**FIG 4:**
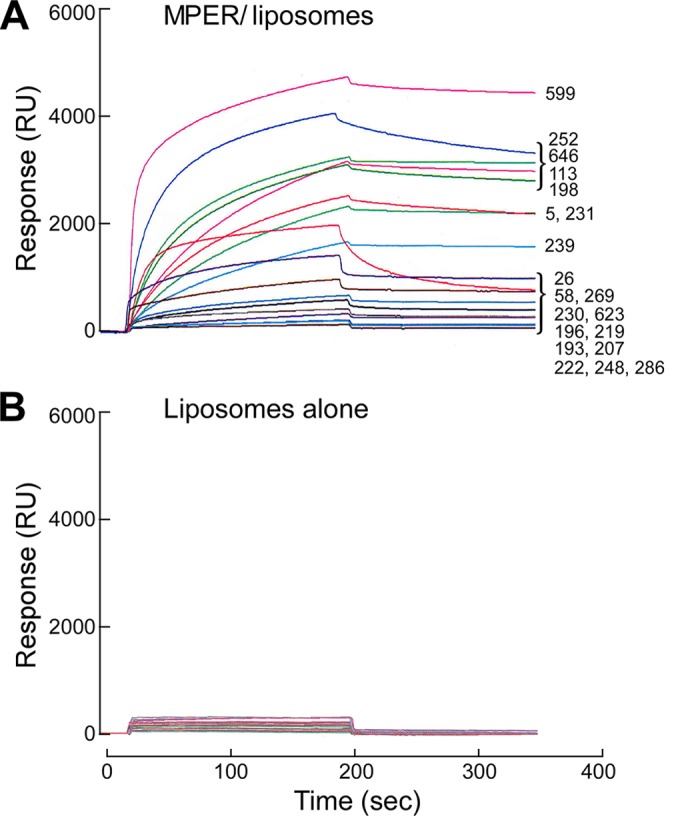
Binding specificities of rMAbs analyzed by SPR analysis. Results are shown for 20 rMAbs binding to MPER/liposomes (DOPC/DOPG) (A) and to liposomes only (B). The scale bars have been equalized to demonstrate the clear specificity for the MPER. Each purified rMAb was tested at 30 μg/ml by injection over L1 chip-bound MPER/liposomes or liposomes only at a flow rate of 10 μl/min for 3 min.

We next selected a representative group of matched IgH/IgLk sequences that included CDRH3 lengths across the range of 5 to 19 amino acids and sequences representing diverse gene families. A total of 20 rMAb clones were selected for expression and characterization by cloning of the selected amplicons into IgH/IgLk expression vectors as described previously (53). The purified rMAbs were then confirmed to have MPER specificity by SPR analysis. Figure 4A and B show relative binding affinities of 20 rMAbs for the MPER arrayed on the surfaces of MPER/liposomes and for liposomes alone. Although LLPC are high-affinity antibody producers, not all MPER-specific rMAbs appear to be high affinity by qualitative measures, as exemplified in Fig. 4A and 1C. While several antibodies showed modest binding reactivity to liposomes alone, the majority of rMAbs had no or weak lipid binding, with no direct correlation to the affinity of the Abs for MPER.

**FIG 5:**
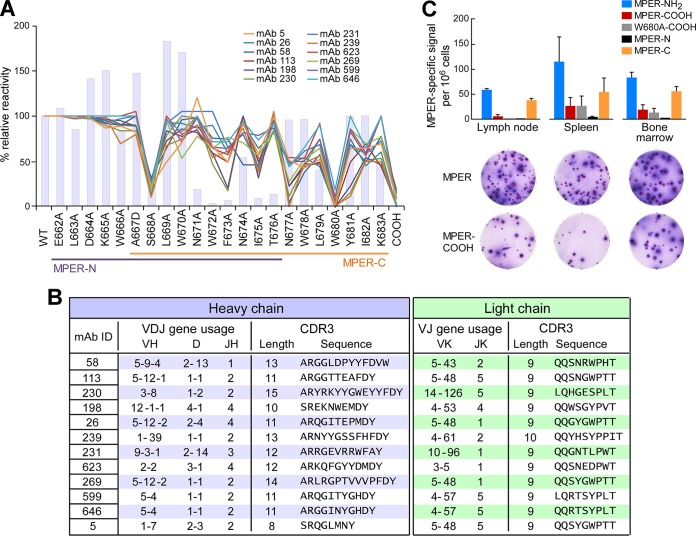
rMAb epitope mapping analysis reveals convergence to a common epitope at the C-terminal region of the MPER and use of various CDRH3 lengths. (A) Epitope specificity analysis by BIAcore of 12 representative rMAbs by use of liposome-bound serial single alanine MPER mutants. The *y* axis shows the percent relative binding activities of Abs against each single-residue mutant compared to that of wild-type MPER (WT) (100%). Colored lines represent the epitope specificities of MPER/liposome immunization-derived antibodies, and bars represent the BNAb 10E8. (B) IgH (blue table) and IgLk (green table) V(D)J gene usage and CDR3 analysis of the 12 representative rMAbs from panel A. The germ line gene segment usage, CDR3 lengths, and sequences of the MPER-specific Abs were determined via IMGT/HighV-QUEST analysis. (C) Frequencies of MPER-specific ASCs with different specificities in various organs 5 days after the second immunization with Npalm-MPER/liposomes. Numbers of MPER-binding ASCs were determined by ELISPOT assay, using each of the different epitope-specific MPER/liposomes as the capture antigen. MPER-NH_2_ and MPER-COOH indicate the HXB2 MPER with C-terminal NH_2_ and COOH ends, respectively, and W680A-COOH indicates a W680 mutation to A with a C-terminal COOH. The MPER-N (E662 to T676) and MPER-C (A667 to K683) sequences are indicated in panel A.

To determine the fine epitope specificity of the rMAbs, we performed SPR binding analysis using a panel of single alanine mutants comprising each of the 22 MPER amino acids. Given that an amide group at the C terminus of the MPER was critical for antibody binding in polyclonal immune sera (42), an MPER-COOH construct was also included to evaluate the binding contribution of the amide. The binding reactivities of all 20 purified rMAbs were reduced by MPER-COOH mutation, indicating that all rMAbs recognized the C-terminal helix of the MPER. Based on the hierarchy of relative binding affinities (Fig. 4A) and the genetic usage of the 20 rMAbs tested, further fine epitope mapping was performed on a collection of 12 rMAbs. As shown in Fig. 5A for the representative set of 12 rMAbs, a conserved antibody binding footprint was observed, requiring S668, N677, W680, and the C-terminal amide in MPER. However, for W672, F673, N674, I675, and L679, each specific residue's contribution to binding differed somewhat between antibodies. Overall, the rMAbs showed fine epitope specificities similar to but distinct from those of 4E10 and 10E8 (6, 12). In addition, with the exception of clones 599 and 646, V_H_ and V_L_ gene usages suggested that all were derived from different germ line B cell lineages with different CDRH3 lengths, ranging from 8 to 15 residues (Fig. 5B), demonstrating that a surprising amount of gene diversity can lead to a conserved MPER epitope specificity.

As an orthogonal approach, we screened the ASCs for epitope specificity by ELISPOT assay. Besides the MPER-COOH mutant noted above, we designed a second amide-lacking variant with a W680A mutation (W680A-COOH). Further, we included two truncated MPER peptides. One, termed MPER-N, contained the amino acids from E662 to T676, and the second, termed MPER-C, included amino acids A667 to K683, comprising the nominal 10E8 binding site. All five of these peptides were N-terminally palmitoylated for ease of incorporation into liposomes. We arrayed them on liposomes and screened ASCs from BM, inguinal lymph nodes (iLN), and spleens for reactivity 5 days after the initial booster immunization with Npalm-MPER/liposomes (Fig. 5C). The MPER-NH_2_ variant, which was the immunogen, represented the most frequent ASC specificity (∼50 iLN, ∼100 spleen, and ∼75 BM ASCs/10^6^ cells) (Fig. 5C). On the other hand, MPER-COOH and W680A-COOH captured antibodies from ASCs at significantly lower frequencies (∼5 iLN, ∼25 spleen, and ∼20 BM ASCs/10^6^ cells for MPER-COOH and ∼0 iLN, ∼25 spleen, and ∼10 BM ASCs/10^6^ cells for W680A-COOH). Furthermore, while MPER-N was not bound by antibodies from ASCs in the iLN, spleen, or BM, the MPER-C was bound. Taken together, these results indicate that although weak, a small fraction of Abs recognize the C-terminal region of MPER independently of the NH_2_ group, and the results are consistent with the antibody titer against MPER-COOH detected in serum (Fig. 1A).

### MPER/liposome immunizations do not generate polyreactivity to cardiolipin and dsDNA.

**FIG 6:**
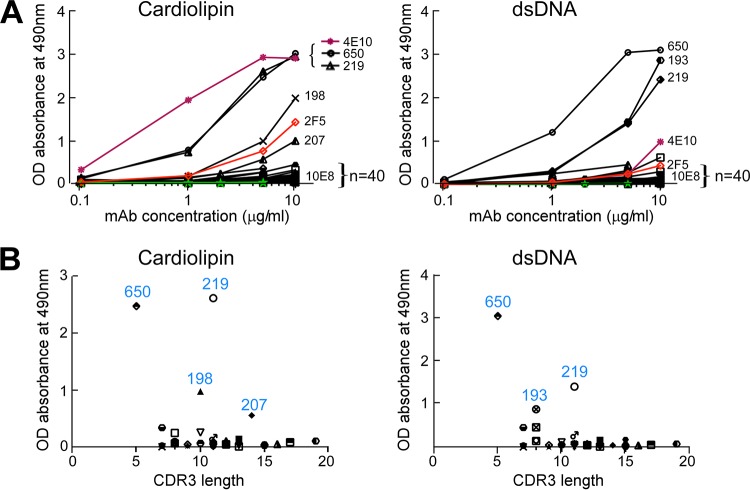
Low frequencies of autoreactivity and polyreactivity of MPER-specific Abs elicited by immunization with various MPER/liposome vaccines. (A) Forty-four different MPER-specific rMAbs from mice immunized with liposome vaccines containing Npalm-MPER, the Npalm-W680A mutant, or Cpalm-MPER were tested for autoreactivity and polyreactivity by cardiolipin and dsDNA ELISAs and were compared with anti-MPER BNAbs (red, 2F5; purple, 4E10; and green, 10E8). Data shown are representative of three independent experiments. (B) Lack of correlation between reactivities and CDRH3 lengths among the 44 different Abs tested in panel A. The *y* axis represents the absorbance at 490 nm of bound antibodies from panel A at 5 μg/ml, plotted against CDRH3 length on the *x* axis.

MPER-specific BNAbs, such as 2F5 and 4E10, have long CDRH3 and have been found to be polyreactive. In addition, both the lipid-integrated nature of the MPER and the amino acid sequence of the MPER shared with some parts of autologous proteins (kynureninase and splicing factor 3B subunit 3) have been proposed to make the MPER stealthy to all but nonpolyreactive antibodies (47). Given that the MPER/liposome-elicited rMAbs all displayed a common binding footprint, but with distinct genetic and biochemical properties, including CDRH3 length (Fig. 5), we reasoned that if polyreactivity were the characteristic of MPER-specific Abs, many antibodies with long CDRH3 would bind to standard model targets, such as cardiolipin and/or dsDNA. In addition to the 20 rMAbs generated with the Npalm-MPER immunogen, another 22 rMAbs, generated from MPER mutant Npalm-W680A/liposome and MPER-Cpalm/liposome (MPER palmitoylated at the C terminus) immunizations, were also tested for cardiolipin and dsDNA reactivity and compared with 2F5, 4E10, and 10E8. Notably, most antibodies (39/44 rMAbs) tested did not bind either cardiolipin or dsDNA (Fig. 6A), including three rMAbs elicited with the MPER mutant Npalm-W680A/liposome immunization, which exhibited 2F5-like epitope specificity. Among the 20 rMAbs characterized in Fig. 4 and 5, four antibodies (198, 207, 219, and 193) were polyreactive. The epitope specificities of antibodies 198 and 230 were distinct but were most similar to that of 10E8 (Fig. 5A), with only antibody 198 manifesting cardiolipin binding. The rMAbs 198 and 207 bound cardiolipin similarly to the BNAb 2F5 but half as well as antibodies 650 and 219 at 5 μg/ml. Antibodies 650 and 219 also bound dsDNA at concentrations down to 0.1 μg/ml and 1 μg/ml, respectively, while rMAb 193 joined them, with dsDNA sensitivity at concentrations down to 1 μg/ml. All five of these rMAbs recognized the C-terminal helix of MPER. The five antibodies that showed polyreactivity (650, 219, 198, 207, and 193) had CDRH3 lengths ranging from 5 to 14 residues. Although a long CDRH3 loop has been associated with polyreactivity (66), no correlation between polyreactivity and the length of the CDRH3 loop was observed in this study (Fig. 6B).

### The CDRH3 loops of MPER-specific antibodies reveal dissociable antigen specificity and polyspecificity properties.

**FIG 7:**
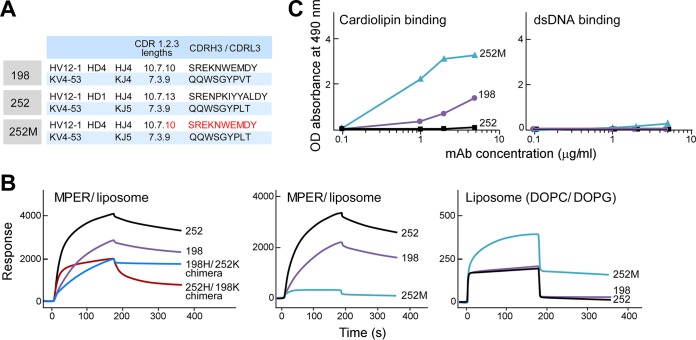
The CDRH3 loop plays a critical role in MPER specificity versus polyspecificity. (A) IgH and IgLk V(D)J gene usage of the related Abs 198 and 252. 252M is a 252 variant in which CDRH3 of clone 252 was replaced by CDRH3 of clone 198. (B) Relative binding affinities of chimeric rMAbs for MPER/liposomes compared to those of WT clones 198 and 252 as measured by SPR analysis (left), binding reactivities of 252M for MPER/liposomes in comparison to those of WT clones 198 and 252 (middle), and lipid-binding reactivities of clones 198, 252, and 252M (right). (C) rMAbs were tested for polyreactivity by cardiolipin and dsDNA ELISAs.

**FIG 8:**
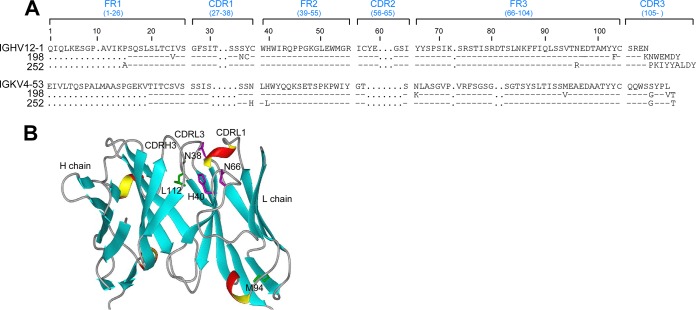
Comparison of rMAbs 198 and 252. (A) Amino acid residue alignments of rMAbs 198 and 252. (B) Modeling of rMAb 198 with predicted IgLk residue differences from rMAb 252. Residues N/H38, H/L40, K/N66, V/M94, and V/L112 were modeled on an immunoglobulin variable region ribbon structure (heavy chain [PDB entry 1ACY] and light chain [PDB entry 5D8J], with homologous sequences). Residues V94 and V112, within FR3 and CDRL3, respectively, are illustrated as the homologous residues M94 and L112 in rMAb 252. Note that N38 and H40, located in CDRL1 and FR2, respectively, are adjacent to the CDRH3 and are replaced by H38 and L40 in rMAb 252. As illustrated, residue N66 at the beginning of FR3 is also near CDRL3 and is K66 in rMAb 198.

To further define the dual reactivity of MPER-specific Abs, we evaluated the biochemical properties of the antigen combining sites of two different sets of antibodies. Clones 198 and 252 shared the same V_H_ gene and J_H_ gene segments (HV12-1/HJ4) and IgLk V_L_ gene usage (KV4-53) but differed in their J_L_ gene usage (KJ4 versus KJ5, respectively) (Fig. 7A). These clones utilized different D gene segments, resulting in very different CDRH3 sequences (SREKNWEMDY for clone 198 and SRENPKIYYALDY for clone 252). Interestingly, the binding properties of these two clones also varied significantly. Clone 252 bound MPER/liposomes with a higher on-rate and a higher overall response than those of clone 198 by SPR analysis (Fig. 7B), but by ELISA, clone 198 bound the lipid cardiolipin, while clone 252 binding was barely detected (Fig. 7C). Therefore, clone 198 reactivity manifested both MPER-specific reactivity and polyreactivity, while clone 252 was MPER specific and lacked polyreactivity (Fig. 6A and 7C). The DOPC/DOPG liposome binding of both clones 198 and 252 was weak (Fig. 7B, right panel), and this trend was supported by negligible reactivity of either clone to dsDNA (Fig. 7C). As shown in Fig. 7B (left panel), when the IgH of clone 252 was paired with the IgLk of clone 198 (252H/198K chimera), binding to MPER was dramatically reduced, with only ∼30% residual binding reactivity relative to that of the clone 252 parent IgG pair. Similar results were shown with 198H/252K chimera binding to the MPER relative to that of clone 198. Next, the chimeric rMAb 252M was made by swapping the CDRH3 of clone 252 with that of clone 198 to determine the critical role of CDRH3 for antigen specificity. Swapping of the CDRH3 of clone 198 with that of clone 252 resulted in the abrogation of 252M binding to MPER/liposomes as determined by SPR analysis (Fig. 7B, middle panel). On the other hand, 252M bound cardiolipin with 3 times the absorbance of its parent CDRH3-containing rMAb 198 and with 10 times the absorbance of clone 252 at a concentration of 5 μg/ml (Fig. 7C). Similar to clones 198 and 252, the chimeric Ab 252M did not have dsDNA reactivity (Fig. 7C). Note that nonspecific liposome binding (DOPC/DOPG) of 252M was also increased compared to that of clones 252 and 198 (Fig. 7B, right panel). As shown in Fig. 8A, the sequences of clone 252 IgL and clone 198 IgL differ by 5 amino acid residues. Two of these residues are relatively homologous, i.e., M94 (clone 252)/V94 (clone 198) in the FR3 region and L112/V112 near the end of the CDRL3 loop, and probably have little effect on MPER binding. H38/N38 is in the CDRL1 loop, which may contact the MPER, and L40/H40 in FR2 is buried but adjacent to the CDRH3 loop (Fig. 8B). It is possible that these two residues influence the conformation of the CDRH3 loop, resulting in the reduced MPER binding observed with the 198H/252K and 252H/198K chimeras (Fig. 7B, right panel). The fifth residue, N66/K66, located at the beginning of the FR3 region, is also in the vicinity of the CDRH3 loop and may play a minor role. The CDRH3 loop in clone 198 contains a tryptophan residue at its apex that confers better membrane binding than that with the CDRH3 loop in clone 252. Interestingly, membrane binding of 252M increased significantly even compared to that of wild-type clone 198, which has the same CDRH3 loop (Fig. 7B, right panel). It is possible that the identical CDRH3 loops in these two constructs adopt different conformations as a result of neighboring residues or that the approach angles of the antibodies with respect to the membrane are different. Notwithstanding this possibility, membrane binding and cardiolipin binding were uncorrelated with MPER binding for the wild-type clones 198 and 252 and the CDRH3 loop-swapped mutant 252M.

**FIG 9:**
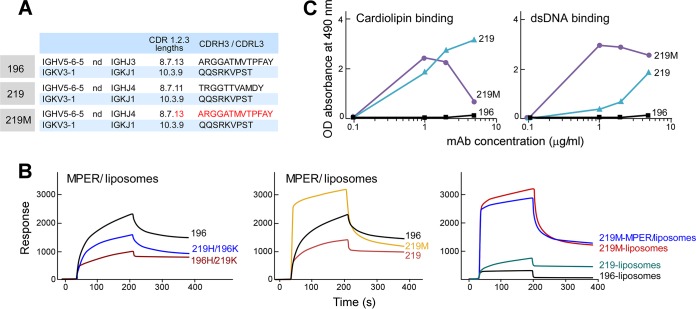
Reverse correlation between MPER binding and polyreactivity determined by chain swap and/or CDRH3 mutations. (A) IgH and IgLk V(D)J gene usage of related Abs 196 and 219. 219M is a 219 variant in which CDRH3 of clone 219 was replaced by CDRH3 of clone 196. (B) Relative binding reactivities to MPER/liposomes as assessed by SPR analysis of chimeric rMAbs from clones 196 and 219 compared to that of naturally paired clone 196 (left panel). Also shown are 219M binding reactivities for MPER/liposomes (middle) and liposomes only (right) in comparison to those of WT clones 196 and 219. Thirty microliters of each antibody at 100 μg/ml was injected over the MPER/liposome or liposome surface of an L1 chip at a flow rate of 10 μl/min for 3 min. (C) rMAbs were tested for polyreactivity by cardiolipin and dsDNA ELISAs.

In a second set of clones, 196 and 219, a trend toward a reverse correlation between MPER binding and polyreactivity was determined by chain swap and/or CDRH3 mutations in the antibody combining site. Clones 196 and 219 share the same V_L_/J_L_ gene pairings (KV3-1/KJ1) and have different V_H_/J_H_ gene pairings (HV5-6-5/HJ3 and HV5-6-5/HJ4) (Fig. 9A), and they differ by 13 IgH and 5 IgLk amino acids. As shown in Fig. 9B, mispairing of IgH and IgLk of clone 196 with those of clone 219 reduced MPER/liposome binding compared to that of clone 196 (left panel). Replacement of clone 219 CDRH3 with that of clone 196 to create a new clone, 219M, greatly increased the MPER/liposome on-rate and total binding (Fig. 9B, middle panel). However, this enhanced MPER/liposome binding was almost entirely due to a nonspecific lipid binding contribution (Fig. 9B, right panel). Along with increased cardiolipin and dsDNA binding by 219M (Fig. 9C), these results suggest that the 196 and 219 antibodies affinity matured toward MPER specificity independently of polyreactivity.

## DISCUSSION

Previously, we characterized the polyclonal immune serum responses of BALB/c mice elicited against an MPER/liposome vaccine incorporating molecular adjuvants and CD4 T cell help (41, 42). In the present study, we focused on defining the LLPC in BM that are responsible for that serum antibody production. The implementation of microengraving methods to assess MPER/liposome specificity at the level of single plasma cells allowed us to investigate their immunoglobulin V_H_ and V_L_ gene usage and to characterize representative rMAbs to elucidate the immunogenicity of MPER/liposomes in finer detail. Utilizing liposome-arrayed MPER immunogens, we observed that the majority of MPER-specific LLPC in BM produce antibodies directed to the C-terminal helix of the MPER region and exhibit evidence of affinity maturation (Fig. 3, 5, and 7). These results imply that these antibodies represent polyclonal immune responses elicited in sera by use of Npalm-MPER/liposomes, as described previously (42). It is evident that LLPC are derived mainly from memory B cells after booster immunization, as they are minimally, if at all, detectable after primary vaccination but readily evident, both serologically and by ELISPOT assay, following booster immunization (Fig. 1 and 5; data not shown). B cell repertoire analysis (Fig. 3) in conjunction with epitope mapping of rMAbs (Fig. 5) collectively indicated that a prominent common epitope specificity is generated by utilization of a variety of different germ line genes. This recognition of antigen follows independent B lineage pathways through affinity maturation, exploiting the structural plasticity of the antigen combining site to achieve this common goal.

Antibody binding to cardiolipin, dsDNA, or Hep-2 cells is commonly measured to assess polyreactivity or autoreactivity. As shown in Fig. 6, only 5 of 44 MPER-specific rMAbs generated by various MPER/liposome vaccines were polyreactive and/or autoreactive. No clear correlation was observed between the CDRH3 length and polyreactivity among the tested mouse MPER-specific antibodies, nor was there an association of polyreactivity with a particular V gene family encoding IgH or IgLk variable regions. When CDRH3 of rMAb 252 was replaced with that of the genetically related clone 198 to create 252M, the MPER epitope specificity was abrogated. Conversely, the 252M reactivities for both cardiolipin and dsDNA were increased compared to those of clone 252. Note that polyreactivity of clone 198, but not clone 252, was observed despite identical MPER epitope specificities and with usage of the same IgH and IgLk V genes by both rMAbs. Similar results demonstrating MPER/liposome reactivity independent of polyreactivity were obtained with a different pair of rMAbs, 219 and 196. These data suggest a critical role of the CDRH3s of both clones 252 and 219 in determining MPER specificity. Given that the MPER binding affinities of the rMAbs are also affected by interchanged pairings of IgH and IgLk, it appears that the fine specificity of anti-MPER rMAbs requires a structural fitness of the antibody combining site shaped by affinity maturation of B cells. In addition, and perhaps more importantly, the results demonstrate that not all MPER-specific antibodies are inherently polyspecific and/or autoreactive: polyreactivity of the MPER-specific rMAbs is separable from antigen specificity in these examples. Our findings are supported by a recent work characterizing a CD4-binding-site-specific BNAb lineage over time which tracked polyreactivity and antigen specificity and found that precursors were capable of obtaining and losing polyreactive characteristics independently of their progression toward the final BNAb (67). Whether the nonspecific liposome binding and polyreactivity independent of MPER specificity of 252M result from increased flexibility of CDRH3 and/or hydrophobicity introduced by sequence changes remains to be tested. In line with these results, increased polyspecificity was observed with alterations of the length and hydrophobic mutations of CDRH3 of 2F5 (29). Mispairings of IgH and IgL among 10E8 variants also increased polyreactivity (68).

The majority of the rMAbs specific for the MPER manifested weak or no liposome binding (DOPC/DOPG), with only several rMAbs showing qualitatively modest lipid binding, including clone 219 (Fig. 4 and 9). In this regard, the recent crystal structure of 4E10 in complex with lipids showed a CDRH1 loop interaction with the lipid head groups and a CDRH3 loop interaction with the hydrophobic acyl chains, emphasizing that lipids appear to be an integral component of the 4E10 epitope (22). Given that the MPER immunogen is embedded in the lipid membrane, it would be interesting to test whether the MPER-specific rMAbs we generated recognize lipids differentially as part of their epitope and the extent to which the lipid interaction observed is linked structurally to polyspecificity as a by-product.

Elicitation of MPER-specific BNAbs has proven difficult. Given the polyreactivity and autoreactivity of 2F5 and 4E10, it has been suggested that immune tolerance mechanisms might limit the elicitation of broadly neutralizing HIV-1 antibodies (47, 50). This hypothesis was further supported by impaired B cell development in knock-in mice expressing the 2F5 or 4E10 V_H_DJ_H_ and V_L_J_L_ rearrangements (69, 70). 2F5 V_H_-V_L_ knock-in mice showed profound deletion of BNAb-expressing B cells in BM at the first tolerance checkpoint, when naive B cells begin to express surface BCR (70). A similar finding was observed for 4E10, for which various mechanisms of negative selection, including receptor editing, clonal deletion, and BCR downmodulation, were noted (69). Human kynureninase and splice factor 3B subunit 3 were identified, through a clever screening strategy, as conserved vertebrate self-antigens recognized by 2F5 and 4E10, respectively (47). Another screen, looking for 4E10 cross-reactivity and spanning a library from the human proteome, did not identify the splicing factor 3B subunit 3 autoreactivity candidate (71). Nonetheless, further support for the 2F5-kynureninase association comes from immunization of opossums, whose kynureninase orthologue lacks the ELDKWA epitope, instead containing the related but distinct ELEKWA sequence. These animals generate high antibody titers to the gp41 2F5 motif, in contrast to mice and other species (47).

While it appears that the aforementioned tolerance mechanisms may preclude or severely limit the generation of BNAbs against the MPER, an increasing number of naturally infected HIV patients produce MPER antibodies, including those with neutralizing activity (6, 72–75). Such antibodies develop without clinical evidence of autoimmune sequelae. Therefore, the difficulty in generating BNAbs directed at the MPER via immunization may relate primarily to issues of immunogenicity. In this respect, our earlier work (42) revealed how immunogenicity is dominated by residue accessibility. Sequence changes or modifications of MPER orientation relative to the membrane easily modulate immune responses and immunodominance. Whereas antibodies with specificity for a 2F5-like epitope were not elicited by the Npalm-MPER/liposome vaccine, 30 to 40% of MPER-specific plasma cells were directed to the N-terminal region of the MPER as assessed by ELISPOT assay when mice were immunized with mutant MPER Npalm-W680A/liposomes or MPERTM/liposomes, the latter of which contain the transmembrane region of gp41 (42; data not shown). Further characterization of rMAbs generated with the mutant MPER Npalm-W680A/liposome vaccine showed that the ELDKWA residues in the 2F5 epitope are critical for antibody binding, including a modest binding contribution from the K and A residues. The observation that MPER/liposome vaccines elicited strong humoral responses in BALB/c mice in our study but not in comparably immunized B6 mice (45) emphasizes how strain differences in inbred mice alter the outcome of the immune response. Our results also highlight that the context of immunogen presentation, including its precise three-dimensional display and sequence, plays a critical role in humoral responses by modulating B cell selection.

The present work reveals that MPER-specific antibodies recognizing a common epitope can be generated from genetically diverse germ line B cells without confinement to a specific gene usage but manifesting different functional characteristics. Some rMAbs exhibit polyspecificity, yet the majority do not share this feature. In keeping with our results, even though the 10E8 epitope overlaps that of 4E10, these MAbs manifest low and high polyreactivities/autoreactivities, respectively (51), and demonstrate different modes of binding (6, 21, 22). The discovery of 10E8 strongly implies that nonautoreactive BNAbs exist and can be elicited. Consistent with this notion, extensive screening of the rMAb 10E8 on an array of >9,400 human proteins showed modest cross-reactivity with only one protein, the intracellularly localized FAM84A protein (51).

We suggest that the polyclonality of B cells responding to the MPER peptide immunogen in a liposome context allows the immune system to generate a productive humoral response. While some of the theoretically possible responders in the repertoire may be removed before they emerge into the periphery due to mechanisms revealed through the B cell developmental studies of 4E10 and 2F5, others lacking polyreactivity/autoreactivity can progress to LLPC. Thus, the MPER target itself in a membrane context will not preclude the generation of protective antibodies. It is also notable that we demonstrate here an ability to derive LLPC comprising several percentage points of the total BM niche, with persistence of antibody production, over a very substantial fraction of the mouse lifetime. These antibodies recognize an epitope mapping to the 10E8 region, spanning the region from S668 to the end of the segment, but unlike 10E8, they are nonneutralizing. The lack of neutralization activity is due in part to the fact that they recognize the artificial C-terminal NH_2_ group (42). Eliminating this C-terminal NH_2_ group dependence while mimicking a more native structural configuration of the MPER likely will demand further modification of the MPER/liposome immunogen and may require inclusion of the transmembrane domain of gp41. Collectively, our data suggest that the greatest challenge to BNAb generation will be the design of vaccines to foster the correct immunogenicity targeting the native MPER exposed on the viral membrane-bound trimer.
